# Persistent pseudobulbar affect secondary to acute disseminated encephalomyelitis

**DOI:** 10.3402/snp.v5.26210

**Published:** 2015-03-18

**Authors:** Zhendong Li, Shijian Luo, Jianying Ou, Rihe Huang, Ying Wang

**Affiliations:** 1 Department of Neurology, The Fifth Affiliated Hospital of Sun Yat-Sen University, Zhuhai, China;; 2 Department of Rehabilitation Medicine, The Fifth Affiliated Hospital of Sun Yat-Sen University, Zhuhai, China; 3 Department of Radiology, The Fifth Affiliated Hospital of Sun Yat-Sen University, Zhuhai, China

**Keywords:** central nervous system disease, emotion, affective lability, pathological laughter and crying, involuntary emotional expression disorder, complication

## Abstract

Pseudobulbar affect (PBA) is a common complication of central nervous system diseases such as stroke, multiple sclerosis, and other neurological diseases, but it remains under-recognized and under-treated in the clinic. PBA caused by acute disseminated encephalomyelitis (ADEM) has rarely been reported. Here, we report a 30-year-old Chinese woman who has experienced PBA from ADEM for 7 years. The patient's principal manifestations were extreme emotions or tears when she saw, heard, or spoke about sad news or other sad things; the durations of these unmanageable emotions were often less than 30 sec, and they occurred at frequencies that ranged from one to several times a day. Occasionally, she laughed uncontrollably while people were talking despite a lack of funny or sad stimuli in the conversation or the surrounding environment. Thus, her social functioning was impaired. This case indicates that the long-term PBA can occur secondarily to ADEM, and this possibility should be considered clinically to ensure timely identification and treatment.

Pseudobulbar affect (PBA) is a common complication of central nervous system diseases, and existing reports have established associations of PBA with stroke, multiple sclerosis, and other neurological diseases (Ahmed & Simmons, 2013; Brooks, Crumpacker, Fellus, Kantor, & Kaye, 2013). Additionally, it has been reported that deep brain stimulation and chemotherapy can also cause PBA (Amtage et al., 2013; Low, Sayer, & Honey, 2008; Min & Khare, 2013; Okun et al., 2004). PBA is characterized by involuntary episodes of crying or laughter that are unrelated or disproportionate to environmental stimuli and to the patient's mood at the time of each episode; crying episodes are more common (Ahmed & Simmons, 2013; Pioro, 2011). The incidence of PBA ranges from 5% to well over 50% across different studies; the overall prevalence has been estimated to be approximately 10% across the six commonly associated neurological conditions in the USA (Ahmed & Simmons, 2013; Work, Colamonico, Bradley, & Kaye, 2011). PBA is referred to by different terms, including pathological laughter and crying (PLC), affective lability (AL) or emotional lability, affective instability, compulsive laughing or weeping, emotional incontinence, emotionalism, excessive emotionality, inappropriate hilarity, involuntary emotional expression disorder (IEED), pathologic emotionality, and other names (Miller, Pratt, & Schiffer, 2011).

PBA can cause patient's severe distress, embarrassment, and social disability; thus, PBA affects the patient's quality of life, quality of relationships, health status, and social and occupational functioning (Colamonico, Formella, & Bradley, 2012). However, the multiple names and insufficient understanding of this condition have led to confusion and misdiagnosis (Ahmed & Simmons, 2013; Brooks et al., 2013; Work et al., 2011).

Acute disseminated encephalomyelitis (ADEM) is an uncommon inflammatory demyelinating central nervous system disease, and survivors of ADEM can exhibit a variety of lasting neuropsychiatric abnormalities that rarely include PBA (Alper, 2012; Parrish & Yeh, 2012). Hoshino et al. (2008) reported a case of AL that occurred in the acute phase of ADEM; here, we report a case of ADEM complicated by long-term PBA.

## Case report

A 30-year-old female, married, Chinese customs declarant with a high school education and employment at a clothing company was admitted to the neurology unit of our hospital on May 15, 2007. She presented with complaints of dizziness and markedly decreased memory for 7 days; for example, she experienced difficulty recalling events from 2 to 3 days ago and forgot commonly used telephone numbers. Simultaneously, she also experienced weakness in her right limbs and discomfort across her entire body. She reported having a cold 2 months prior to admission. Her personal and family histories were normal. On the day of admission, her general physical examination was normal, and a neurological examination revealed reduced memory of recent events, reduced calculation ability, right limb paresis, active left biceps and bilateral knee reflexes, positive Babinski's sign on the left, and no other abnormalities. She became increasingly sick over the next 4 days and successively exhibited dysarthria, irritability, indifference, lack of spontaneous speech, negativism, poor comprehension, occasional compulsive weeping when asked about her condition, and two generalized tonic–clonic seizures. Moreover, her right upper limb muscle strength decreased to a level of severe impairment, her limb tendon reflexes became hyperfunctional, and she exhibited a forced grasping reflex on the left, bilateral Rossolimo's sign (dominant on the right side), bilateral Babinski's sign, Gorden's sign on the right, and an obviously stiff neck.

**Fig. 1:**
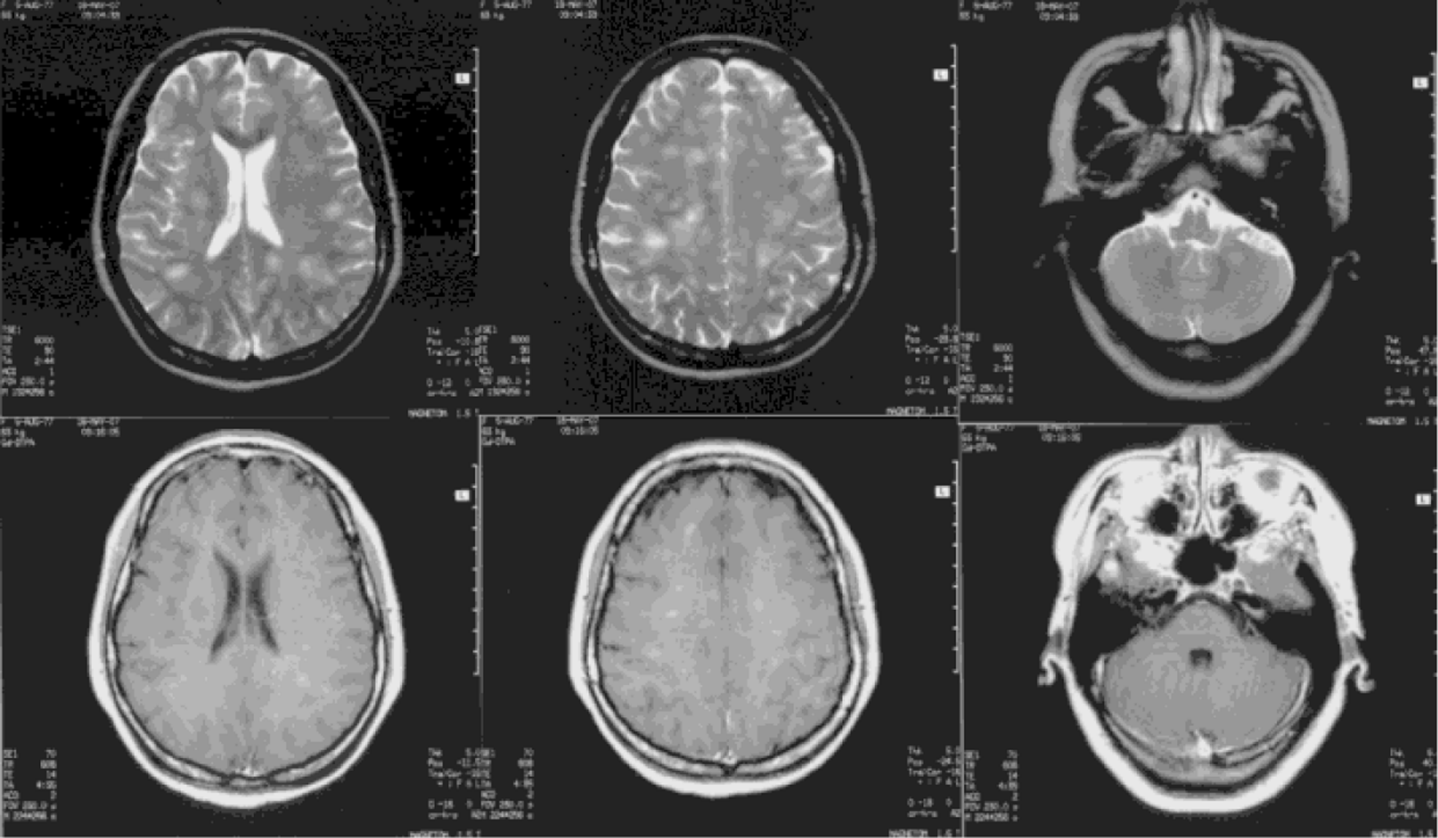
A brain MRI on the 10th day after disease onset: various sized patches of slightly long T1 and slightly long T2 signal intensities scattered across the bilateral parietal gray–white matter junction zones, the subcortical white matter, and the semi-oval center and left cerebellum. Some of these lesions had clear boundaries, and others indicated brain swelling; most of the lesions exhibited small patchy enhancements following the intravenous injection of Gd-DPTA.

Blood investigation revealed that her full blood count; liver and kidney functions; electrolyte, glucose, lipid, and coagulation function; erythrocyte sedimentation rate; and immunologic tests were all normal. Cerebrospinal fluid analysis was normal. Electroencephalography revealed a widespread mild to moderate abnormality. A brain MRI on the 10th day after disease onset revealed various sized patches of slightly long T1 and slightly long T2 signal intensities scattered across the bilateral parietal gray–white matter junction zones, the subcortical white matter, and the semi-oval center and left cerebellum. Some of these lesions had clear boundaries, and others indicated brain swelling; most of the lesions exhibited small patchy enhancements following the intravenous injection of Gd-DPTA (Fig. 1). Her temperature changed from 36°C to 37.4°C during her hospitalization.

**Fig. 2:**
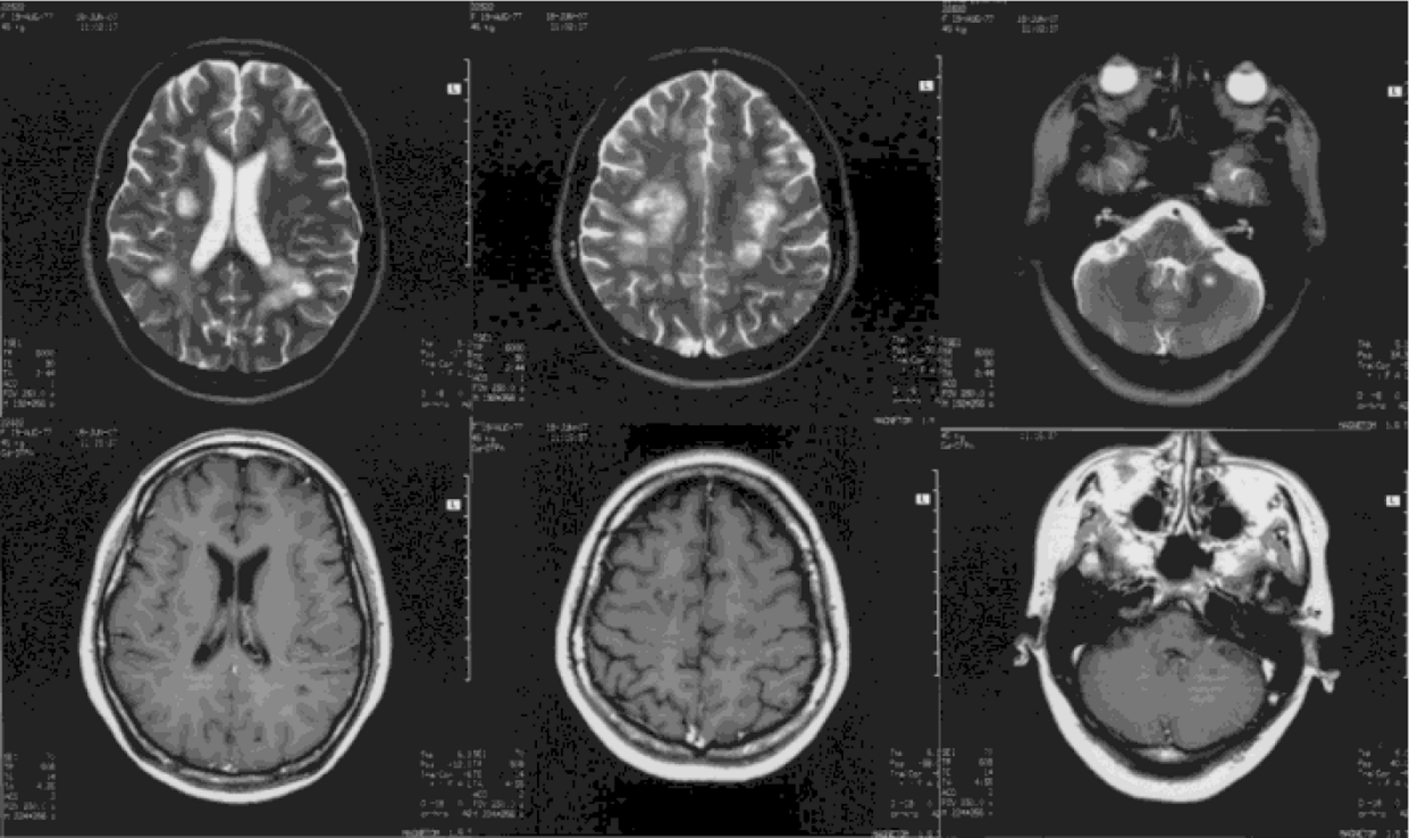
In the 5th week after disease onset, repeated brain MRIs: the original lesion had become more clear, the brain swelling had disappeared, most of the lesions exhibited no enhancement, and individual lesions with mild small patchy enhancements remained present but with significantly reduced signal intensities.

On the 12th day after disease onset, she accepted pulse therapy with methylprednisolone (1 g/D, 5 days) and immunoglobulin (0.4 g/Kg/D, 5 days) and a subsequent tapering oral dose of prednisone (60 mg/d, 2.5 months). Her condition gradually recovered. In the 5th week after disease onset, repeated brain MRIs revealed that the original lesion had become more clear, the brain swelling had disappeared, most of the lesions exhibited no enhancement, and individual lesions with mild small patchy enhancements remained present but with significantly reduced signal intensities (Fig. 2). Other than mildly impaired calculation ability, slight euphoria, occasional compulsive weeping, mild dysarthria, slightly increased right upper limb tension, limb tendon reflex hyperfunction, bilateral ankle clonus, positive right Babinski's sign, and Gorden's sign, the patient's other symptoms and signs had disappeared during the 7th week after disease onset, and then she was discharged.

**Fig. 3:**
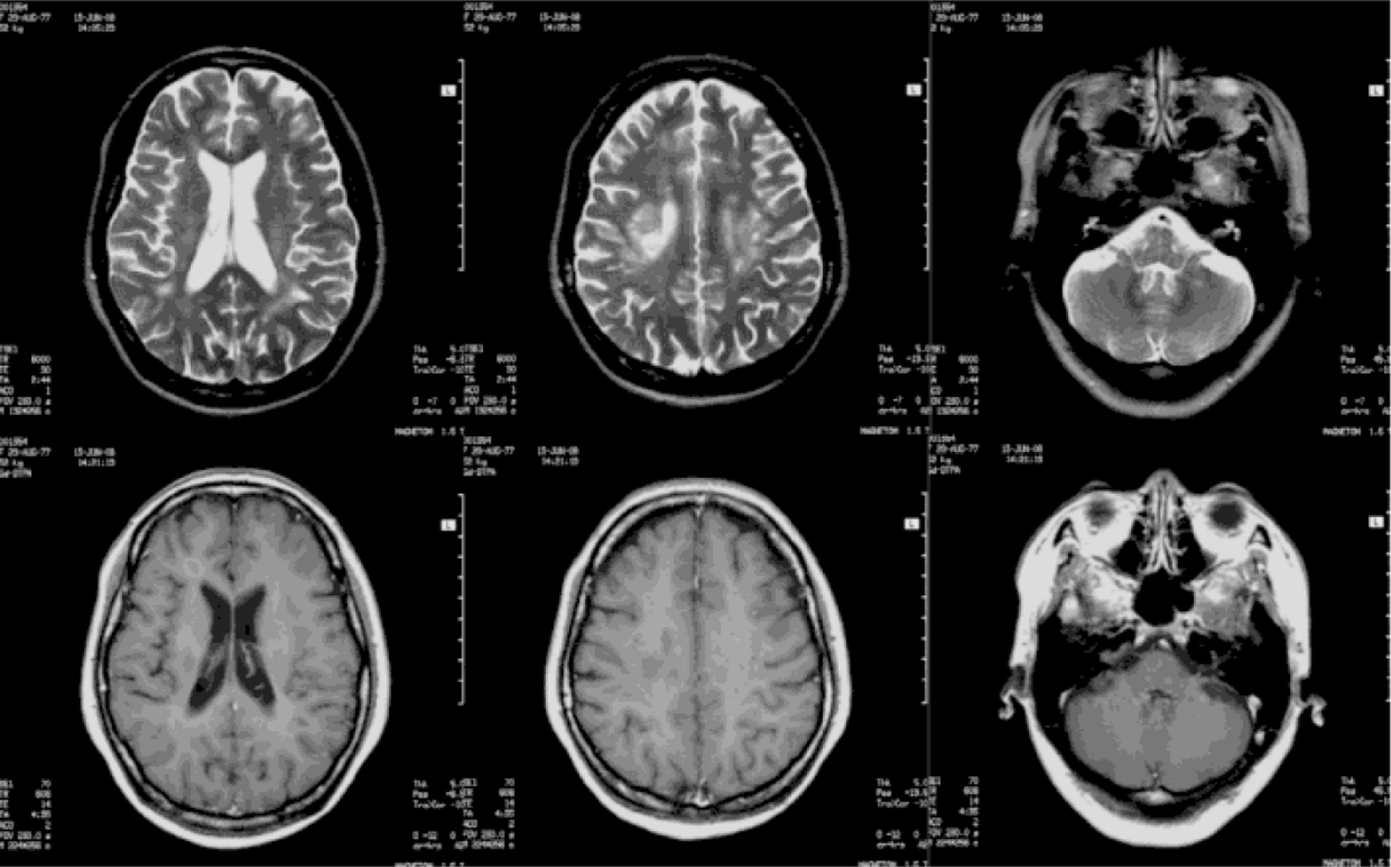
Brain MRIs 1 year after the disease: the lesion number was similar to that originally observed, but the signal intensities of the lesions had significantly decreased, the boundaries were clear, and lesions exhibited no enhancement.

**Fig. 4:**
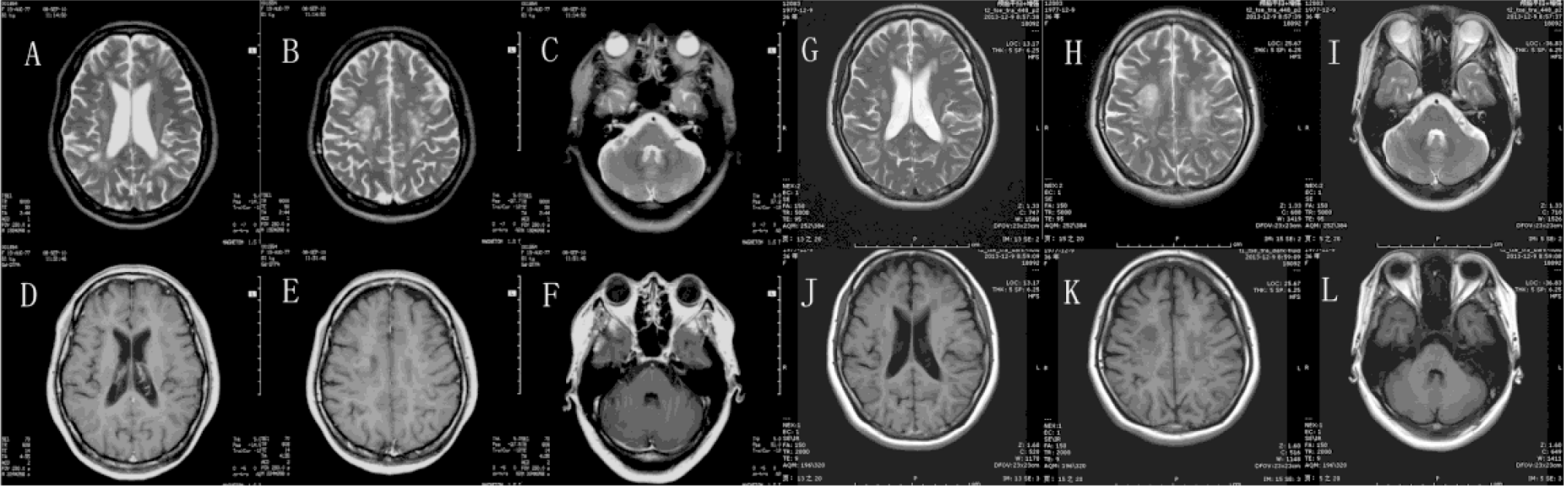
Two repeated brain MRIs revealed no new changes. A, B, C, D, E and F were scanned in September 2010. G, H, I, J, K and L were scanned in December 2013.

The patient underwent a follow-up interview 1 year after the disease. In addition to experiencing nonfluent speech and a fatigable right leg, she frequently experienced difficulty controlling her emotions during her recovery period at home after discharge. She became emotional or began to sob when she saw, heard, or spoke of minor sad news or events, for example, while watching TV related to or talking about disasters and misfortune. The durations of these uncontrolled emotions were frequently less than 30 sec, and they occurred between once and several times a day. Occasionally, she laughed uncontrollably when people were talking despite the lack of a funny or sad stimulus in the conversation or the surrounding environment. Initially, the condition often caused her family or friends to feel strange, but they gradually adapted to her behavior. She returned to work 6 months after discharge. However, due to difficulty in communicating and interacting with people, she quit work 1 month later and became a full-time housewife. The episodes of emotion continued. Neurological assessments at this time revealed difficulty initiating pronunciation, a positive Babinski's sign on the right, and no other signs. Repeated brain MRIs revealed that the lesion number was similar to that originally observed, but the signal intensities of the lesions had significantly decreased, the boundaries were clear, and lesions exhibited no enhancement (Fig. 3). Her condition was diagnosed as ADEM with PBA. The dysarthria was improved by language training, but due to concerns about the potential drugs harm to her regarding pregnancy, the patient refused treatment for PBA. The PBA had continued over the next 6 years, and two repeated brain MRIs revealed no new changes (Fig. 4). A follow-up assessment in the 7th year after the disease onset revealed slight difficulty in initiating pronunciation, a positive Babinski's sign on the right, a Western aphasia battery (WAB) score of 98.5/100 (Kertesz, 2007), a Hasegawa's Dementia Scale – Revised (HDS-R) score of 29/30 (Kato et al., 1991), and Hamilton Depression Scale (HAMD) and Hamilton Anxiety Scale (HAMA) scores of 0 (Clark & Donovan, 1994; Hamilton, 1967). Additionally, her Center for Neurologic Study-Lability Scale (CNS-LS) score was 17/35, and her Pathological Laughter and Crying Scale (PLACS) score was 16/54 (Moore, Gresham, Bromberg, Kasarkis, & Smith, 1997; Robinson, Parikh, Lipsey, Starkstein, & Price, 1993). On these two latter tests (CNS-LS and PLACS), crying was the symptom primarily responsible for the score. Her Social Functioning Exam (SFE) score was 5/56, which indicated mild social dysfunction related to her sex life, job satisfaction, and use of social services (Starr, Robinson, & Price, 1983).

## Discussion

The patient's primary disease met the International Pediatric Multiple Sclerosis Study Group criteria for ADEM; thus, the final diagnosis was ADEM (Krupp et al., 2013). Survivors of ADEM can exhibit continued cognitive dysfunction, epilepsy, cranial nerve damage, and sensory or motor disabilities (Alper, 2012; Parrish & Yeh, 2012). However, in addition to the continued slight dysarthria and motor disorders, our case was complicated with long-term episodic emotional expression disorder. The CNS-LS and PLCS are more objective scales for the measurement of PBA, and their cutting scores are all ≥13 (Moore et al., 1997; Robinson et al., 1993). The two scale scores in our case were respectively 17 and 16, which supported the diagnosis of PBA, and the SFE assessment revealed impairments to the patient's life and work.

PBA is thought to include two subtypes, that is, PLC and AL (Miller et al., 2011). In PLC, the expression of emotion is involuntary and uncontrollable; expressed emotions are exaggerated relative to both the stimulus and the current emotional state. Emotional expression can also be incongruent with the current emotional state regarding valence (Miller et al., 2011). For patients with AL, expressions can be partially controllable and expressed emotions can be exaggerated relative to the stimulus, but such emotions are congruent with the subjective (internally experienced) emotional state (Miller et al., 2011). Lauterbach et al. (2013) differentiated the two subtypes more clearly. PLC has at least three characteristics: 1) stimuli that are often inadequate in intensity or inappropriate in emotional valence trigger responses of laughing or crying (e.g. sad stimuli trigger laughing); 2) laughing and/or crying often are independent of or do not always correspond to the patient's mood immediately prior to the laughing and/or crying behavior; and 3) the laughing and/or crying behavior is stereotyped in that the same responses are exhibited each time (e.g. if inappropriate laughter or crying is exhibited, all inappropriate responses are laughter or crying) regardless of the stimulus (Lauterbach et al., 2013). In addition to these three characteristics, PLC entails that each occurrence of the laughing and/or crying behavior exhibits at least one of the following stereotyped features regardless of the stimulus: each episode is of the same intensity (i.e. severity), the same duration, or the same frequency (Lauterbach et al., 2013). AL involves the following three characteristics: 1) stimuli might be inadequate in intensity but are appropriate in emotional valence for triggering laughing or crying responses (i.e. sad stimuli trigger crying but not laughing, and pleasant or humorous stimuli trigger laughing but not crying); 2) laughing and/or crying correspond to the patient's mood state immediately before the laughing and/or crying episode (i.e. patients who were happy do not cry tears due to sadness, and those who were sad do not laugh) unless a valence-congruent stimulus is overwhelming; and 3) the laughing and/or crying behavior is not stereotyped (i.e. the emotional displays vary from episode to episode in terms of intensity, duration, or frequency of occurrence) (Lauterbach et al., 2013). In our case of persistent long-term PBA, the main manifestations were crying episodes in response to sad stimuli and occasional episodes of laughter, which accorded with the characteristics of the AL subtype.

Although the concept of PBA was proposed more than 100 years ago, its pathogenesis remains unclear (Lauterbach et al., 2013). For a long time, it was believed that the laughing and crying center in the upper brainstem losing the inhibition by the motor cortex induced PBA, and thus, PBA was a loss of inhibition or release phenomenon (Wilson, 1924). Progress in research revealed that the occurrence of PBA is related to complex structures, including the cerebral cortex, white matter, basal ganglia, diencephalon, limbic system, brainstem, and cerebellum. These structures compose the volitional and emotional pathways of emotional expression; damage to the volitional pathway leads to PLC, and damage to the emotional pathway leads to AL. Furthermore, the emotional pathway is regulated by the volitional pathway, and the pathways involve a variety of neuromodulators, including serotonin, glutamate, dopamine, acetylcholine, etc. (Lauterbach et al., 2013; Parvizi et al., 2009). In our case, the primary damage occurred in the bilateral parietal gray–white matter junction, the subcortical white matter (predominantly on the left side) and the left cerebellar white matter; thus, damage to the emotional pathway might have been involved in the resultant AL.

Based on the above-mentioned pathophysiology, the clinically recommended treatments for PBA are primarily tricyclic antidepressants, selective serotonin reuptake inhibitors, and the cough suppressant dextromethorphan/quinidine (Ahmed & Simmons, 2013; Miller & Panitch, 2007; Miller et al., 2011; Pioro, 2011). Emerging evidence has revealed that the antipsychotic drug aripiprazole, the antiepileptic drug valproic acid, and traditional Chinese medicine are also effective (Johnson & Nichols, 2014; Magaudda, Imbesi, & Di Rosa, 2012; Zhou & Li, 2014). However, unfortunately, our patient refused further treatment due to concerns about the effects of drugs on pregnancy; thus, the PBA continued for an extended period and exhibited its natural profile.

PBA is easily confused with mood disorders, such as depression, and attention should be given to the differential diagnosis (Miller et al., 2011). In addition to clinical manifestations, some scale instruments can help identify. The HAMD and HAMA were respectively used to evaluate depression and anxiety, and their cutting scores are all ≤7 (Clark & Donovan, 1994; Hamilton, 1967). Based on the HAMD and HAMA scores of 0, the depression and anxiety were excluded in our case. Additionally, the WAB (cutting score < 93.8) was for the measurement of aphasia and HDS-R (cutting score ≤20/21) for the measurement of dementia (Imai & Hasegawa, 1994; Kertesz, 2007). The two conditions were also excluded based on the scale assessments in our case. The terminological confusion surrounding PBA is also notable and is not conducive to the identification and study of this clinical phenomenon; thus, unifications of the terminology and diagnostic criteria are needed. Cummings et al. (2006) proposed the use of IEED and the corresponding diagnostic criteria, but this proposal remains to be accepted. Because the term PBA is used most frequently and PBA is caused by organic brain damage that typically involves bulbar paralysis, we believe that the term PBA is easiest to understand and should be accepted.

## Conclusion

At present, PBA remains clinically under-recognized and under-treated. Our case indicated that long-term PBA secondary to ADEM can impair patients’ social function, and clinicians should be aware of this condition to ensure timely identification and treatment.
